# Temporal population structure, a genetic dating method for ancient Eurasian genomes from the past 10,000 years

**DOI:** 10.1016/j.crmeth.2022.100270

**Published:** 2022-08-22

**Authors:** Sara Behnamian, Umberto Esposito, Grace Holland, Ghadeer Alshehab, Ann M. Dobre, Mehdi Pirooznia, Conrad S. Brimacombe, Eran Elhaik

**Affiliations:** 1 Department of Biology, Lund University, 22362 Lund, Sweden; 2 Department of Animal and Plant Sciences, University of Sheffield, Sheffield S10 2TN, UK; 3 Department of Automatic Control and Systems Engineering, University of Sheffield, Sheffield S1 3JD, UK; 4 National Heart, Lung, and Blood Institute (NHLBI), Bethesda, MD 20892, USA; 5 Department of Anthropology and Archaeology, University of Bristol, Bristol BS8 1TH, UK

**Keywords:** genomics dating, ancient DNA, temporal population structure, TPS, genomic dating, paleogenomics, phenotypic traits, DNA-based dating method, radiocarbon dating, supervised learning, random forest regression

## Abstract

Radiocarbon dating is the gold standard in archeology to estimate the age of skeletons, a key to studying their origins. Many published ancient genomes lack reliable and direct dates, which results in obscure and contradictory reports. We developed the temporal population structure (TPS), a DNA-based dating method for genomes ranging from the Late Mesolithic to today, and applied it to 3,591 ancient and 1,307 modern Eurasians. TPS predictions aligned with the known dates and correctly accounted for kin relationships. TPS dating of poorly dated Eurasian samples resolved conflicting reports in the literature, as illustrated by one test case. We also demonstrated how TPS improved the ability to study phenotypic traits over time. TPS can be used when radiocarbon dating is unfeasible or uncertain or to develop alternative hypotheses for samples younger than 10,000 years ago, a limitation that may be resolved over time as ancient data accumulate.

## Highlights

• ∼50% of ancient genomes lack reliable dates, which leads to poor understanding
• Genomic data contain markers indicative of the age of the sample
• TPS is an AI-based genomic dating tool that converts DNA data to dates (<10,000 BP)
• TPS can be used to date ancient genomes and resolve dating disputes

## Motivation

Accurate dating is essential to the interpretation of paleogenomic data. The gold standard method in archeology is radiocarbon dating. However, a major limitation of radiocarbon dating is the high amount of collagen extraction involved in the process. Consequently, almost half of all published ancient genomes lack reliable and direct dates, which results in obscure and contradictory reports. Here, we present the temporal population structure (TPS), a machine learning-based genomic dating method for genomes ranging from the fringes of the Late Mesolithic to modern times.

## Graphical abstract

**Figure:**
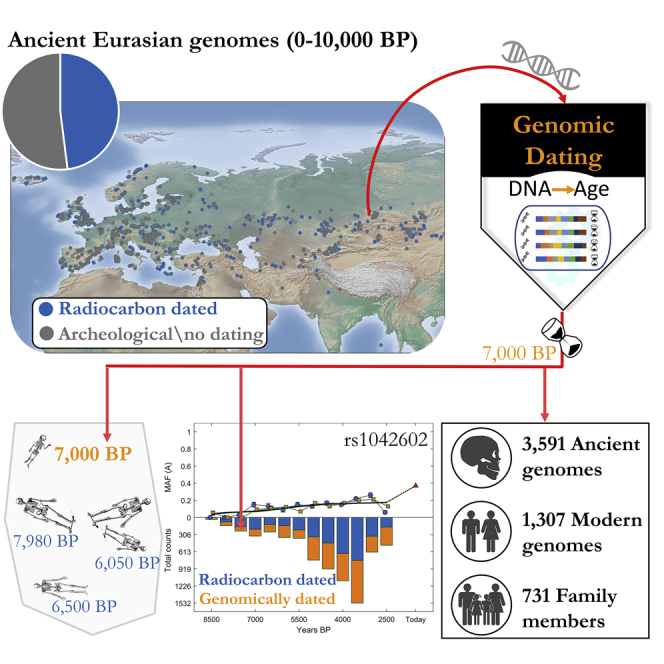

## Teaser

Accurate dating is essential to the interpretation of paleogenomic data, yet nearly half of all ancient genomes lack reliable dates, which results in obscure and contradictory reports. To overcome these problems, Behnamian et al. develop TPS, a genomic dating tool for genomes (<10,000 BP), and demonstrate its accuracy on ∼5,000 genomes.

## Introduction

Ancient DNA (aDNA) has transformed the study of human demographic history, allowing us to directly analyze patterns of past genetic variation rather than infer them post-factum. In the last few years, we have witnessed a conspicuous increase in the volumes of ancient skeletal DNA and studies attempting to trace their origins (Morozova et al., 2016). Dating ancient remains is crucial to producing meaningful and reliable historical reconstructions, particularly in light of the growing medicalization of the field.

In the second half of the 20th century, radiocarbon dating dramatically changed archeology (Libby et al., 1949) and became the gold standard for dating ancient organic materials (Taylor and Bar-Yosef, 2014). Radiocarbon dating is based on the observation that living beings exchange ^14^C with their biosphere while alive and cease to do so when dead. At that point, their ^14^C atoms decay into ^14^N with a half-life of ∼5,700 years, whereas their ^12^C concentration remains constant (Ramsey, 2008). Assuming that the initial ratio of carbon isotopes in the biosphere remained constant over time, measuring the ^14^C–^12^C ratio allows inferring the age of the sample. Over the past 80 years, many improvements to the original method were made (e.g., Ramsey, 2008), including pretreatment of the bones of the samples to eliminate contamination by recent carbon (Jacobi et al., 2006) and the introduction of accelerator mass spectrometry (AMS), which advanced the measurement of the decaying process (Brock et al., 2010). In addition, knowledge of Earth’s past environment and the quantification of reservoir effects and paleodiets further improved the calibration curves of the past biosphere isotope levels (Alves et al., 2018; Ascough et al., 2005; Kromer et al., 2001; Ramsey, 2008). For instance, the bones recovered from Repton (England) were first associated with the Viking Great Army from 873 to 874 CE (1190–1205 years before present [YBP]) based on the archeological context. However, early radiocarbon results predated some of them to the 7th and 8th centuries CE (Biddle and Kjolbye-Biddle, 2001). Only in a later radiocarbon dating that considered the marine reservoir effect were all of the remains consistent with a single late 9th century event, in line with the numismatic evidence (Jarman et al., 2018).

Despite this progress, the dating of ancient remains is fraught with challenges. A major limitation of radiocarbon dating is its requirement for a large amount of collagen. The routine AMS requires at least 60–200 mg of bone (Cersoy et al., 2017), depending on the protein preservation and the extraction protocol, with some labs requiring at least 500 mg with an optimum amount of 1,000 mg. However, even the lesser amounts exceed the collagen available in small vertebrates and remain with a patrimonial value (e.g., hominid remains, bones). For instance, the Repton Viking Army site was dated to 1400–1600 BP (10 mg collagen) and 1250 BP (60 mg collagen), with the higher yield dates being closer to the true values (Bronk Ramsey et al., 2004). Only 50% of the ∼6,500 ancient skeletons whose aDNA was sequenced and published were radiocarbon dated (IntCal20 or SHCal20), and over 10% of the Allen Ancient DNA Resource (AADR) V50 (https://reich.hms.harvard.edu/allen-ancient-dna-resource-aadr-downloadable-genotypes-present-day-and-ancient-dna-data) dates include various warnings. The remaining skeletons have either been dated according to the archeological materials found alongside the sample or remain undated. The subjective interpretation of skeletal data has already led to misunderstandings on numerous occasions. For instance, a bone from the Darra-i-Kur cave in Afghanistan, initially assumed to be from the Paleolithic (30,000 YBP) (Dupree et al., 1972) and often cited as one of the very few Pleistocene human fossils from Central Asia, was recently radiocarbon dated to the Neolithic (4,500 YBP) (Douka et al., 2017). Similarly, one of the Brandýsek site individuals (RISE569) was initially attributed to the Bell Beaker period (4,800–3,800 YBP) (Allentoft et al., 2015), but a later radiocarbon dating post-dated it (1,400–1,100 YBP) (Olalde et al., 2018). Reevaluations of ^14^C calibration curves are not rare (Manning et al., 2018). Not only do different tissues produce different results, but labs may produce radiocarbon ages that differ up to and over 1,000 years (Higham et al., 2006). Contamination is a major problem with radiocarbon dating that leads to erroneous dates. Talamo et al. (2021) showed that in an ancient bone sample (42,000 years old), adding 1% of modern carbon resulted in an 8,000-year shift to a younger age. This bias is illustrated in the case of a human skull from Zlatý kůň in Czechia, which was initially radiocarbon dated to ∼15,000 YBP (Svoboda et al., 2002), redated to ∼27,000 YBP, and again redated to ∼19,000 YBP after the same bone was treated. A protocol that aimed to free contaminating carbon provided a fourth date of ∼34,000 YBP (Deviese et al., 2018) (reviewed by Prüfer et al., 2021). Adding to these technological complications, dates recorded in the AADR continuously change without documentation of the historical changes (275 samples were redated between V44.3 [https://reichdata.hms.harvard.edu/pub/datasets/amh_repo/curated_releases/index_v44.3.html] and V50.0). For instance, sample MA2195, initially dated to 3800 BP (V44.3) was redated to 217 BP (V50) based on the context in both cases and without an explanation. Remarkably, in all cases of date changes, the original publication was cited. Notably, family members, dated initially to different times, are post-processed to appear closer. As misattributions can lead to erroneous conclusions, the uncertainties in the age of nearly half of the aDNA samples and the actual age of the remaining half pose a considerable risk of misinterpretation to the field, which calls into question the cost-effectiveness and overall usefulness of paleogenomic studies.

Genomic dating has tremendous potential to improve paleogenomic studies. Compared with radiocarbon dating, DNA analyses require less material (x1/5) (Korlevic et al., 2018) and can be used to directly date skeletal aDNA for which no radiocarbon date is available or as an independent validation approach for existing results. Previous efforts focused on dating samples based on the idea that Neanderthal ancestry decays over time: Moorjani et al. (2016) reported a correlation between sample age and Neanderthal ancestry for five ancient human genomes (45,000–12,000 YBP) and caution that the correlation is strongest over an age range of 20,000–30,000 YBP and lost for more recent dates. However, this limited cline was likely due to the small cherry-picked dataset since other studies report no appreciable change in Neanderthal ancestry over the last 40,000 years (Petr et al., 2019; Svensson et al., 2021). Moreover, most ancient genomes are younger than 10,000 YBP (Figure S1), with many non-Europeans.

A second method exploited the idea that introgressed fragments, broken down by recombination, become progressively shorter over time. This approach was used to date the controversial Zlatý kůň (Prüfer et al., 2021). Unfortunately, both the measurement of ancestry and estimates of fragment size tend to use *D* statistics (*ABBA-BABA*, *f*3, and *f*4), which have attracted several criticisms. First, *D* is a relative test and always compares the proportion of archaic ancestry in one population relative to another. With recent reports that even Africans carry non-human ancestry (Chen et al., 2020; Povysil and Hochreiter, 2016), it is hard to see how *D* can generate absolute values. Second, *D* statistics and related measures rely on the assumption that the mutation rate is constant, yet this assumption appears false in the face of several reports of mutation rate variation between human populations (Amos, 2021; Harris, 2015; Harris and Pritchard, 2017; Mallick et al., 2016). Third, supporting the idea that mutation rate variation can present a problem, Amos (2021) reported that positive *D* is dominated, not by heterozygous sites in non-Africans, as expected under introgression, but by heterozygous sites in Africans. This finding is consistent with a signal driven by recurrent mutations, not Neanderthal ancestry. Fourth, it has been pointed out that the population substructure can generate patterns virtually identical to those expected from “Neanderthal ancestry” (Eriksson and Manica, 2012); and while debate continues (Yang et al., 2012), this possibility remains a potential confounding factor that should not be ignored. Finally, *D* statistics are extremely simplified models that make many unrealistic assumptions; in addition to single, discrete episodes of gene flow from Neanderthals to humans, they also assume a lack of Neanderthal ancestry in Africans and complete panmictic ancestral populations while ignoring the effects of genetic drift over time (Gopalan et al., 2021). As an alternative to summary statistics like *D*, faith is placed in the inference of introgressed haplotypes to estimate archaic ancestry (reviewed by Gopalan et al., 2021). Unfortunately, this is no less problematic because current approaches fail to include several important real-life complexities, such as mutational non-independence, mutation hotspots, a correlation between mutation rate and recombination rate, and more. These complexities are ignored, not least because our understanding has not reached a level at which the key parameter values are known. Thus, despite the great potential of genomic dating, too many issues have yet to be resolved for these methods to be considered reliable. Overall, using Neanderthal ancestry to date genomes remains unsubstantiated to be deemed reliable, leaving the potential of genomic dating unfulfilled.

## Results

### The temporal population structure (TPS) model

**Figure 1:**
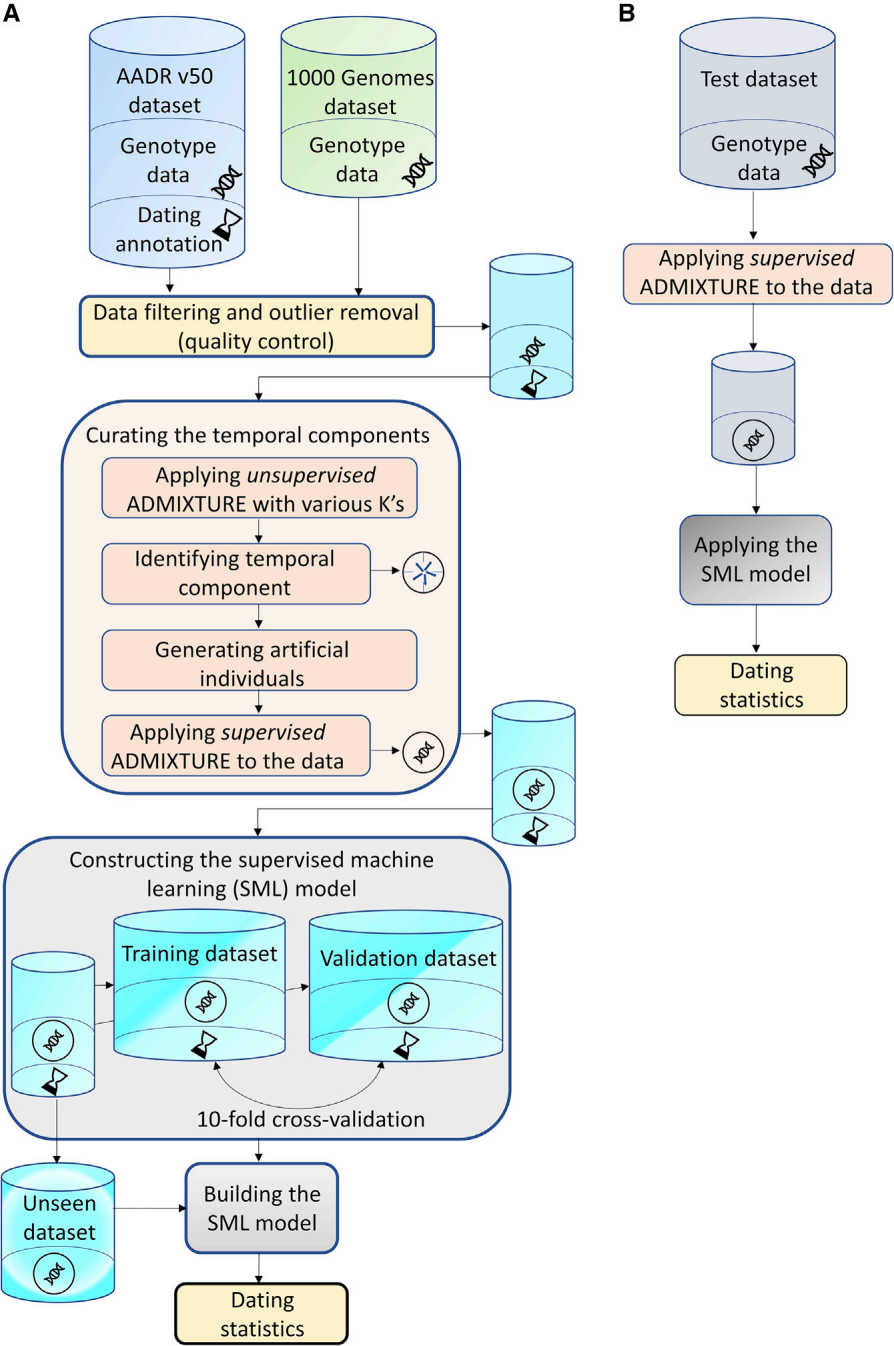
TPS workflow Schematic overview of the dating workflow with TPS (A) and how to apply it to date genomes of unknown dates (B). The SML model created in (A) can be applied to genomic data of the same species (B).

We present the TPS tool, the first DNA-based dating method suitable for Eurasian genomes younger than 10,000 YBP (Figure 1A). The rationale of TPS is that because most human variation is within continental populations (Elhaik, 2012) and is subjected to processes such as selection and genetic drift that modulate the allele frequencies over time (Graur et al., 2013), there exist markers that exhibit substantially different allele frequencies between different periods, irrespective of geography, that can be used to estimate temporal trends. We called these markers time informative markers (TIMs). Conceptually, TIMs are reminiscent of ancient ancestry informative markers (aAIMs) that vary over space (Esposito et al., 2018), except that they operate along the time axis. Whether through natural selection or genetic drift, the changes in allelic frequencies over time create unique allelic combinations across multiple loci that characterize the historical period (not place) when individuals lived. We called these allele frequencies combinations temporal components. Due to their association with time, temporal components can be harnessed to convert genomic data into time and predict the age of a sample solely from genotype data.

**Figure 2:**
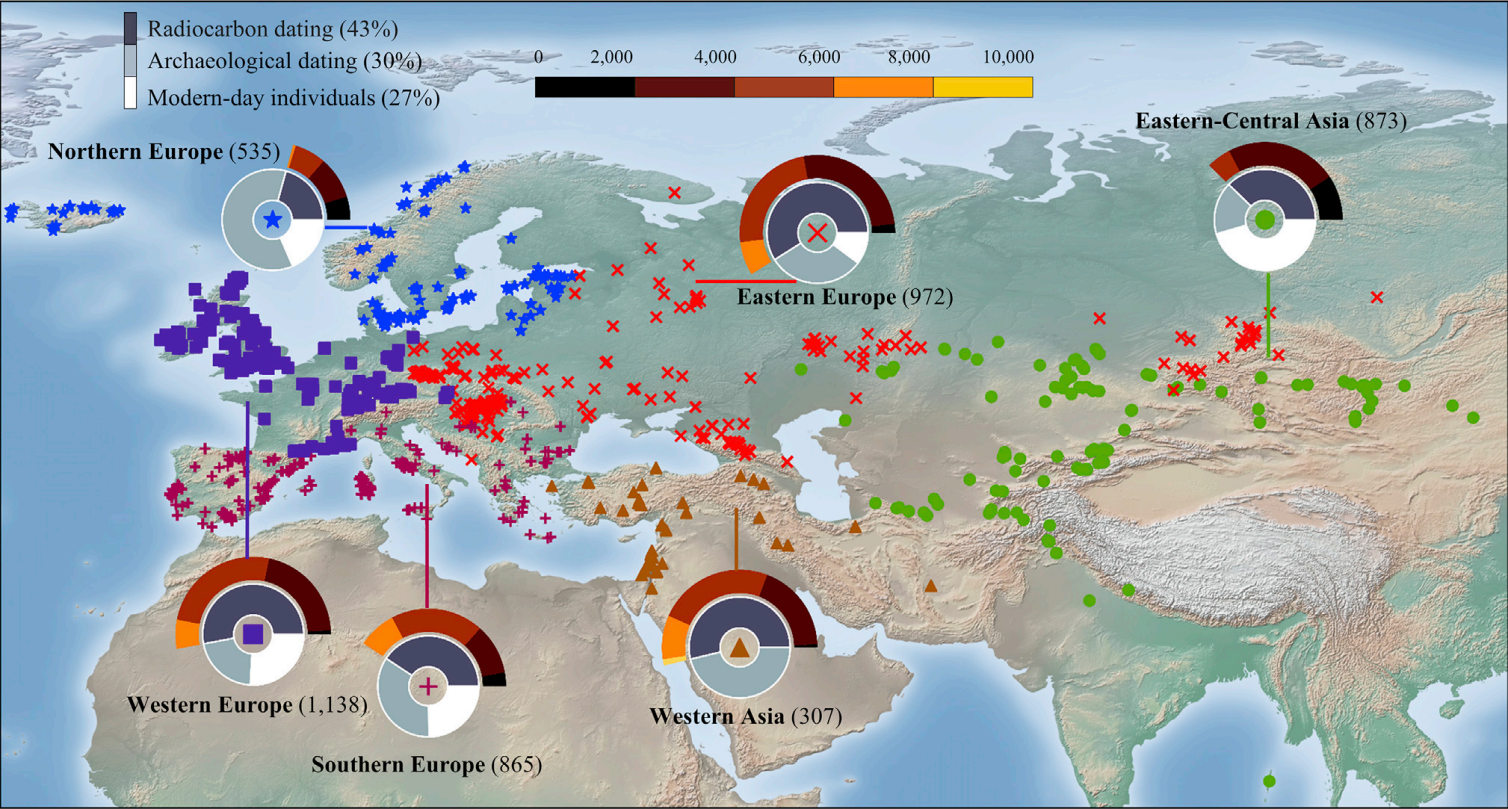
Location and dating of the ancient samples used in this study Symbols mark geographical macro-areas where samples were found. Samples are shape- and color-coded by the region. Sunbursts depict the regional dating annotation of the samples. The inner sunburst shows the proportion of ancient samples dated with different dating methods and modern samples. The outer sunburst marks the distribution of radiocarbon dates for the ancient samples. Radiocarbon dates are divided into 5 temporal bins of 2,000 years (top bar), covering the timeline of our database.

To demonstrate this, we first curated a dataset of ∼5,500 ancient Eurasian genomes (Figure 2; Table S1) ranging from the Mesolithic to the 19th century from the AADR (V50). The genotype data of the samples in this public compendium consisted of single-nucleotide polymorphisms (SNPs) from a panel of ∼1.24 million known informative positions but with high missingness. The major challenges for modeling temporal allelic shifts are the inherent sparsity of the archaeogenetic data and the uncertainties associated with genotyping and dating. For that, we selected ∼150,000 markers with the least missingness and applied quality control procedures (see Method details), after which 3,591 Eurasians from the Late Mesolithic to the 19th century (10,000–90 YBP) remained. Of these, ∼60% were directly radiocarbon dated ($DB_{RD})\;\text{and}$ ∼40% were indirectly dated based on archeological context ($DB_{OD})$. We supplemented this dataset with 1,307 modern Eurasians from the 1000 Genomes Project.

**Figure 3:**
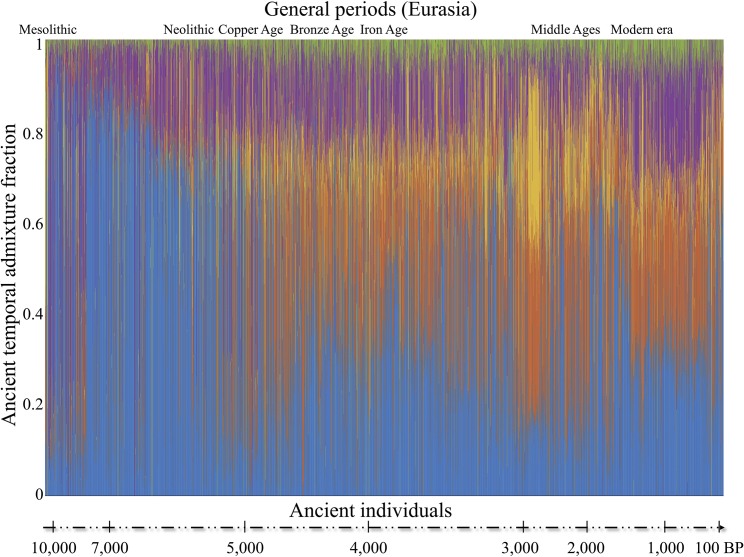
Ancient temporal components for the radiocarbon-dated samples (*DB*_*RD*_) over time The 3 modern temporal components are meager for these samples and were omitted for coherence. Samples are sorted by their age (note that the time x axis is non-linear). Each vertical stacked bar represents an individual. Colors correspond to the 5 ancient temporal components. The plot demonstrates that the temporal components are continuous over time and can be used for genomic dating. Related to Table S2.

Building on our previous approach to representing samples as combinations of genomic components to allow analyzing samples on even grounds (Elhaik et al., 2014), we sought to identify temporal components, generate artificial genomes representing those components, and then represent each ancient genome as a combination of these components (Figure 1A, “curating the temporal components”). For that, we merged a random subset of 300 ancient genomes (Table S2) with 250 modern samples from Europe, Asia, and Africa and applied unsupervised ADMIXTURE (Alexander et al., 2009) with a various number of *K* components (Figure S2A). Five ancient and three modern temporal components captured temporal trends not reflective of ancestry or geography (Figure S2A). Using the allele frequencies of the temporal components, we simulated the DNA of putative “temporal populations” with individual genomes that represented the typical allele combinations of the temporal components (Figure S2; Table S2). Finally, we applied a supervised ADMIXTURE analysis to all of the samples against the temporal populations to calculate the proportion of SNPs associated with each component per sample (Figure 2; Table S2). As expected, we found that each temporal component predominates a delimited time interval (Figure 3) and that temporal samples exhibit similar patterns irrespective of geography (Figure S3).

TPS uses a supervised machine learning (SML) approach, a learning model that uses genomic data (temporal components) to date samples using random forests (Figure 1A, constructing the SML model). The random forest algorithm builds on the concept of a decision tree. A decision tree uses a tree-like structure (or flowchart) in which each internal node is a test, and branches represent the outcome of the test, which leads to other nodes until the end nodes (or leaves) with the outcome are reached. Random forest is based on an assemblage of decision trees, randomly and independently generated based on a subset of the features found in the input data (temporal components) that produce the most separation between the features. Because each tree in the forest “grows” from a random set of features, the trees are diverse and uncorrelated. The output of the random forest is determined by the vote-of-majority of all of the trees to reduce the risk of an inaccurate prediction by individual trees (Breiman, 2001). The SML learns the rules for producing correct answers (dates) from the experience of training on random subsets of the input data. Random forest algorithms are considered robust to noise, fast, scalable, and accurate in life sciences. They also require little parameter tuning, can solve the issue of data overfitting and account for non-linearities in data (e.g., Aguiar-Pulido et al., 2021; Boulesteix et al., 2012; Cutler et al., 2012; Touw et al., 2013; Ziegler and König, 2014). Ensemble learning algorithms like random forests are, therefore, appropriate for medium to large datasets like ours.

We evaluated the accuracy of TPS by training the model on two portions of the samples (validation and training sets) using 10-fold cross-validation. We then applied the model to the third portion of the samples (unseen set) and compared the difference between their TPS predicted and reported dates (Figure 1B).

### Identifying TIMs

**Figure 4:**
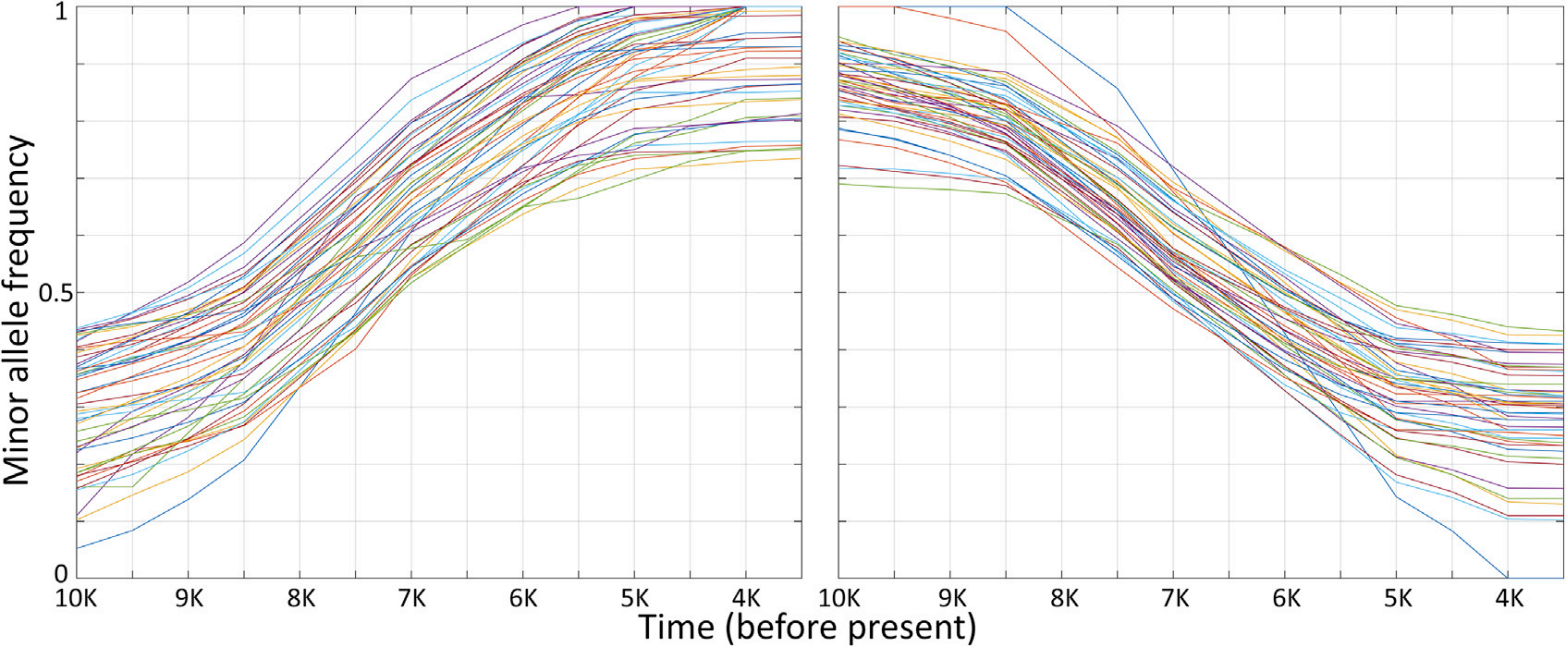
Time series of minor alleles frequencies for the top 100 TIMs that showed the most pronounced 50 increasing (left) and 50 decreasing (right) trends

To identify the genomic markers that underlie the temporal components used by TPS, the temporal components were sorted from oldest to youngest (Figure S4), creating a temporal variation profile of allele frequencies for every SNP. A time-series analysis (see Method details) identified 62,371 SNPs whose allele frequencies either decreased or increased over at least 3,000 years (Figure 4), which we called TIMs. Non-TIMs were SNPs whose allele frequency exhibited little or no variation over time (Table S2). Most of the TIMs (76%) are intronic, intergenic, and non-coding (McLaren et al., 2016); 50% of the coding variants are missense variants. The annotation of TIMs was nearly identical to the annotation of the entire SNP set. To avoid omitting samples due to the high missingness of the original dataset, we used the entire SNP set for the remaining analyses.

### Evaluating the accuracy of TPS

**Figure 5:**
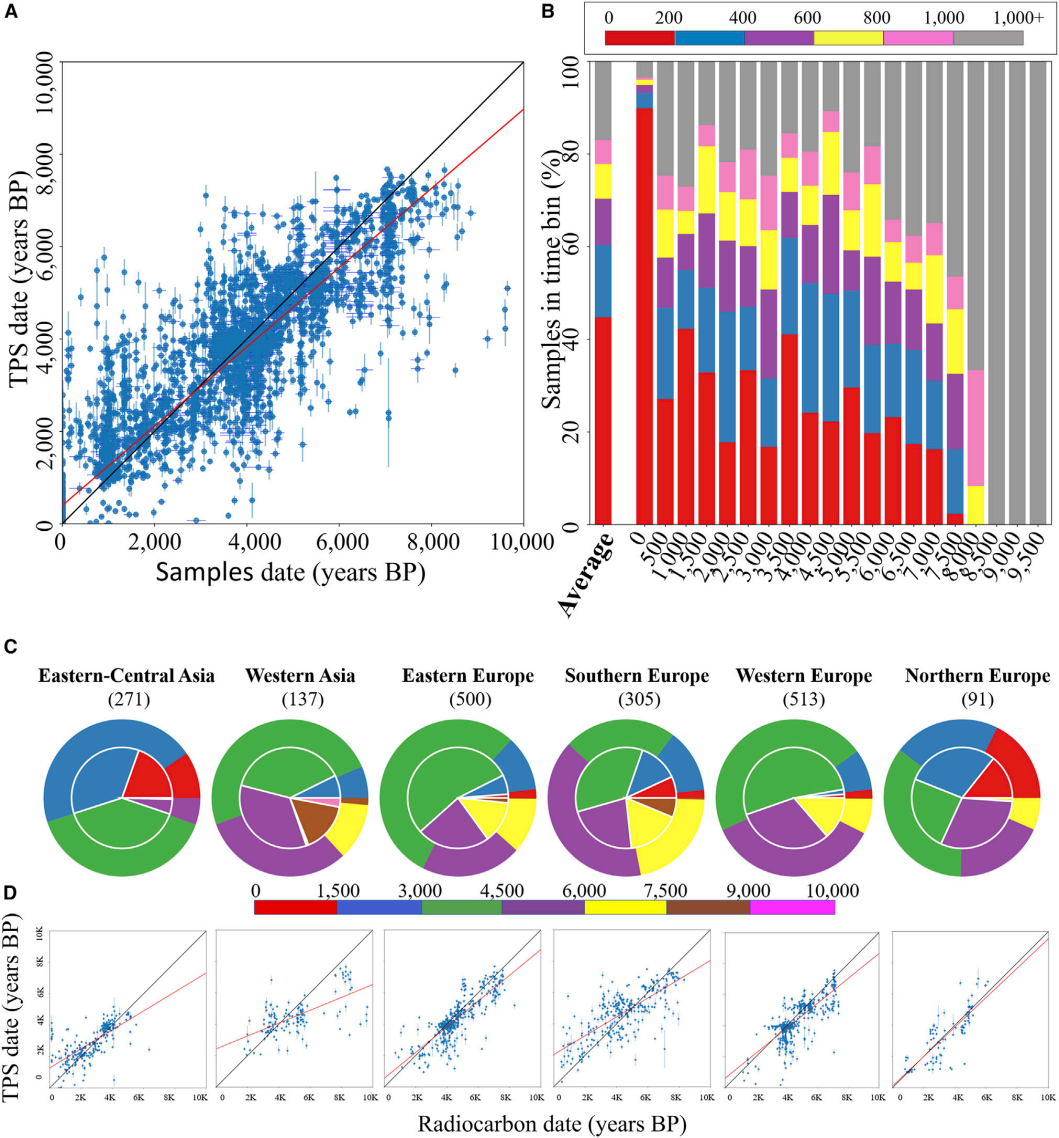
Evaluating the accuracy of TPS dating for ancient and modern samples using the entire SNP set (A) The correlation between TPS and published dates (t test, n = 4898, *r* = 0.93, p value = 0). Vertical and horizontal bars represent the SD of TPS and radiocarbon dating, respectively. The red line represents the linear fit against the y=x line (black). (B) TPS aggregated accuracy. Samples are sorted into 500-year-period bins according to their mean published dates (BP) (*x*-axis) (e.g., the 4,000 YBP bin represents samples dated from 4,000 to 4,499 YBP). Colors reflect the prediction accuracy, calculated as the difference in years between TPS prediction and the sample date. A total of 60% and 70% of the samples were predicted within 400 and 600 years from their published dates, respectively. The prediction accuracy of regional radiocarbon-dated samples is shown in (C) and (D). (C) Contrasting TPS and radiocarbon dates for ancient samples by region. Samples are split into 1,500 years and dated by TPS (outer pie charts) and radiocarbon (inner pie charts). TPS accuracy can be visualized by the overlap of the 2 circles. The number of samples per region is noted. (D) The correlation between TPS and radiocarbon dates for the same samples as in (C) (t test; 91 < n < 513, 0.63 < r < 0.82, 3.72∗10^−168^ ≤ p ≤ 3.65 × 10^−23^). SD bars and the red lines are as in (A).

Next, we compared the known dates of the ancient and modern samples with their TPS-predicted dates (Table S3A). The two dates were significantly correlated (t test, n = 4898, *r* = 0.93, p value = 0). Similar results were obtained when we repeated the analysis for radiocarbon-dated (t test*,* n = 2,137, *r* = 0.79, p = 0.007) and archeological-dated samples (t test*,* n = 1454, *r* = 0.86, p = 0.04). To gauge the reliability of TPS predictions, we defined the accuracy per sample as the absolute difference between its TPS result and its mean radiocarbon or archeological dates. TPS median accuracy for all ancient and modern samples was 259 years, 0 years for the modern samples, and 428 years for ancient samples, with 75% of the samples being assigned a TPS date within 445 years from their radiocarbon date. Only 816 (16%) of the samples were TPS dated over 1,000 years from their mean date (Figure 5B). The general uniformity in the accuracy across the different periods suggests the absence of temporal biases toward any particular period, except the oldest samples for which performance is below average, most likely due to their small sample size.

To gauge the geographical effects on TPS accuracy, we selected the radiocarbon-dated samples from Table S3A, divided them into six regions (Figure 5C), and compared TPS and radiocarbon dates (Table S3A). The two dates were significantly correlated in every region (t test: 91 ≤ n ≤ 513, 0.63 ≤ *r* ≤ 0.82, 3.72∗10^−168^≤ p ≤ 3.65 × 10^−23^) with close alignments to the ideal bisecting line (Figure 5D). Exceptionally high accuracies were found among eastern Europeans, western Europeans, and eastern central Asians (TPS accuracy = 323, 384, and 345 years, respectively), whereas TPS median accuracy for western Asians, southern Europeans, and northern Europeans was lower (804, 774, and 650 years, respectively).

Evaluated on the same ancient and modern dataset as above, TPS accuracy for non-TIMs was 339 years (t test, n = 4,976, *r =* 0.9, p = 0) (Figures S5A and S5B; Table S3B), and 279 years for TIMs (t test, n = 4,893, *r* = 0.91, p = 0) (Figures S5C and S5D; Table S3C). Because the results were comparable to the full set of SNPs (Figures 5A and 5B) and to avoid dropping samples, we continued analyzing the latter.

TPS predictions of same-country samples did not cluster around a single period. Instead, they were spread over the timeline following their radiocarbon dates, confirming that the temporal components represent temporal rather than geographical variation (Figure S5K). TPS accuracy was not correlated with the genomic coverage (two-sided t test, t statistic = −35.31, the two-tailed p = 6.7 × 10^−240^), indicating that TPS is robust to the high missingness common to aDNA data (within the limitation of the 600 SNPs used).

We contrasted the accuracy of TPS against two controls. First, we generated a random matrix of 4,898 × 10 with random values [0,100], associated them with the real dates, applied the TPS model training as in Figure 1, and measured the prediction accuracy (Table S3F). TPS median accuracy was 1,996 years, 2,790 years for the modern samples, and 1,530 years for the ancient ones, with 75% of the samples being assigned a TPS date within 2,800 years. Most of the samples (∼80%) were TPS-dated over 1,000 years from their mean date (Figures S5E and S5F). Second, we carried out a principal-component analysis (PCA) by projecting the ancient DNA samples onto the top 10 PCs defined by modern-day populations (Lazaridis et al., 2016) (Table S3G). As before, we applied the TPS model training to the 4,898 × 10 dataset and measured the prediction accuracy. TPS median accuracy for all of the samples was 592 years, 0 years for the modern samples, and 1,053 years for the ancient ones, with 75% of the samples being assigned a TPS date within 1,632 years from their radiocarbon date. Many samples (38%) were TPS dated to over 1,000 years from their mean date (Figures S5G and S5H). We note the existence of data leakage in the PCA application due to the projection of the ancient samples onto the modern ones, which inflates its prediction accuracy.

To further evaluate the performances and characteristics of TPS, we applied it to three subcohorts as follows. First, we TPS dated 414 relatives from 130 ancient families originally dated with mixed dating methods and then post-processed (AADR, V50) them to reduce disparities between family members (Table S3D). Here, we calculated the difference between TPS dates and other dates and the age difference among family members, who should be dated to the same period regardless of the actual date. Each family was analyzed separately. We adopted a simple post-processing approach for families larger than two, in which the age of samples predicted outside the 30th–70th percentiles of the median age of all samples would be the median of that age. TPS median accuracy for pre- and post-processed results was 348 and 225 years, respectively. TPS median age difference among families for pre- and post-processed results was 283 and 17 years, respectively, compared to the 68 years of post-processed dating (Figure S5L) of the AADR. Post-processed TPS dates also had a significantly lower median and narrower distribution than the AADR dates (two-sided Wilcoxon rank-sum test, p = 2.65 × 10^−64^). TPS median accuracy for 318 relatives from 145 modern families (dated to 10 BP) was 0 years (Table S3D).

Second, we TPS dated modern samples from 13 Eurasian populations (Table S3A). The results were consistent with their modern origins (n = 1,307, TPS accuracy = 0, $\bar{TPSaccuracy}$ = 26, 95% confidence interval [CI] 27 ± 6.02, SEM = 6; all of the units are in years).

**Figure 6:**
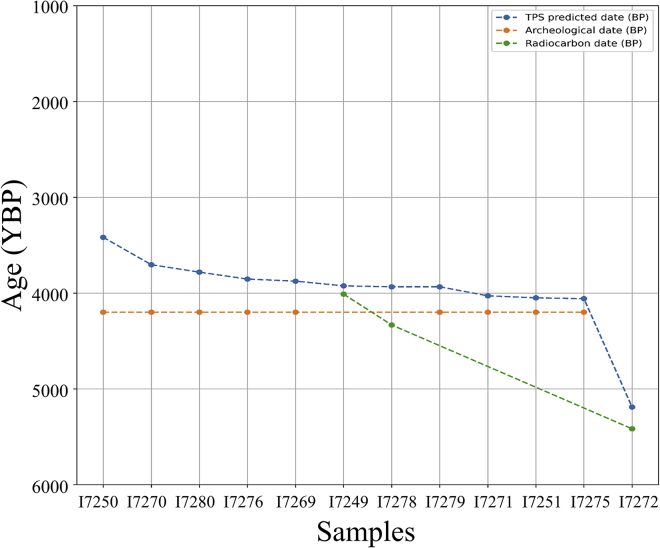
Comparing the TPS and alternative dates for 12 Brandýseks samples TPS dating generally agreed with both radiocarbon and archeological dates.

Finally, to test whether TPS can resolve discrepancies in the literature, we TPS dated the Brandýsek individuals from Czechia. Two individuals (RISE569 and RISE568) from the Brandýsek site were originally attributed by archeological context to the Bell Beaker period (4,800–3,800 YBP) (Allentoft et al., 2015). After they were redated based on radiocarbon and archeologically associated materials, respectively (Olalde et al., 2018), they were removed from the analysis of the Brandýsek individuals (4,850–4,150 YBP) by the latest authors as they post-date the Bell Beaker culture. Excepting these two samples, which TPS excluded as outliers, TPS dates for the remaining 12 Brandýsek individuals showed high similarity to the radiocarbon and archeological dates. The questionable date of individual I7272 (radiocarbon dated to 5,417 YBP) was also confirmed by TPS (5,292 YBP) (Figure 6).

Two observations are noteworthy for these individuals. First, 11 of the remaining 12 individuals were TPS dated to fit within the Bell Beaker and Corded Ware period (4,850–4,150 yBP). Second, the last individual, I7272, was much older and predated the Corded Ware culture, as is evidenced by two additional features: First, I7272 lacked the ancient temporal component present in all of the other individuals at this site, which is ubiquitous among Bell Beaker samples and associated with the period following the Yamnaya invasion (ancient temporal component 2) (Table S3A). Second, the Y haplogroup of I7272 is I2, whereas all of the other males at that site, including the other two attributed to Corded Ware, are R1. Haplogroup R1 dominates post-Yamnaya migration populations (Freeman et al., 2020; Myres et al., 2011; Underhill et al., 2015), while I2 is primarily associated with Paleolithic and Neolithic Europe (Fu et al., 2016; Mathieson et al., 2015). TPS dating, temporal components, and Y haplogroup suggest that I7272 is related to an earlier Neolithic occupation at this site. Moreover, the site consists of architectural features that are not usually associated with Bell Beaker burials, such as the use of stone in graves (Olalde et al., 2018).

### Evaluating the robustness of TPS

We carried out five additional analyses to evaluate the robustness and stringency of TPS to erroneous or noisy input data and mismatching training data. In the first analysis, we tested to what extent samples with identical temporal components but incorrect dates affect the accuracy of the age prediction. For that, we divided the ancient samples (9,647–90 BP) into 10 roughly equal-sized bins (332–382 samples per bin) by their ages and countries. We randomly sampled 740 samples stratified by age and geography and considered unseen samples. Training TPS on the remaining dataset yielded a median base accuracy of 164 years for these 740 samples. We copied these samples to the training set and added 1,000 years to their ages. Their temporal components remained unchanged. Retraining the model on the original training set and the biased samples yielded a median accuracy of 298 years for these samples. Repeating the process after increasing the bias by 2,000 years yielded a similar median accuracy (314 years) (Figure S6A). We thereby showed that (1) TPS predictions become worse when identical samples with biased ages are used in training, although the prediction error is of the same magnitude as the median accuracy of TPS, (2) the prediction error does not increase linearly with the bias, but is roughly capped at approximately twice the number of years from their originally predicted age, and (3) the random forest regression is more robust to dating errors compared to methods based on sequence similarity that would have reported much higher errors.

In our second analysis, we tested the effect of introducing noise to the temporal components of the samples, such as can be generated due to genotyping errors. For that, we used the 740 samples for the unseen test (base median prediction accuracy of 164 years) (Figures S5I, S5J, and Table S3E). We found that even at a maximum noise level applied to 10% of the samples, the accuracy decreased slightly to 199 years (Table S4A). Only when the noise was applied to all of the samples had the median prediction accuracy decreased to 873 (100% noise) years. These results demonstrate the robustness of TPS to biased genotype data.

In our third analysis, we evaluated systematic biases in the 20 studies that published the most ancient samples by TPS dating them separately and comparing the TPS prediction accuracy (Figure S6B). Whereas most of the top 10 studies were well predicted with high TPS accuracy (584 years), the following 10 studies were more poorly predicted (840 years), mainly due to three publications (de Barros Damgaard et al., 2018; Fernandes et al., 2020; Marcus et al., 2020). To understand why these datasets were poorly predicted, we followed up with the same scheme described before, with the 740 unseen samples with a median base accuracy of 164 years. When we excluded the samples of Fernandes et al. from the unseen dataset, the TPS median accuracy improved to 129 years. When we added all of their samples to the unseen dataset, TPS median accuracy decreased to 338 years. After excluding their samples from the training set, retraining the model, and repeating the calculation, the accuracy remained unchanged. A similar trend was found when repeating this procedure for the two other datasets. In other words, although they were poorly predicted, these samples were useful for training the TPS model and predicting the dates of other samples. To understand why this is the case, we plotted the average standard deviation (SD) of the mean age (BP) of samples from each study against their TPS-predicted accuracy (Figure S6C). We found a significant positive correlation (n = 20, *r* = 0.52, two-sided t test, t statistic = 2.59, p = 0.018), suggesting that using the mean age of samples with a wide SD of the age is more challenging for TPS; however, when embedded with other training samples and after filtering samples with a high SD of the mean date (see Method details), they positively contributed toward TPS prediction accuracy.

In our fourth analysis, we evaluated the ability of TPS to date samples when the training dataset lacks samples of comparable ages. For that, we divided all of the samples into 20 windows of 500 consecutive years by their ages. Then, for each age group separately, we dropped the samples of that age window from the training set and predicted the age of the unseen samples after training TPS on the remaining windows. We report the median predicted age (Figure S6D). The overall weighted mean TPS accuracy was 847 years and, as expected, was lower in the extreme windows and higher in the middle windows. The mean TPS accuracy was 1,031 for an analysis of 10 windows of 1,000 consecutive years. These results demonstrate the robustness of TPS to age mismatches between the training and unseen datasets.

In our final analysis, we evaluated the effect of geography on TPS predictions by excluding geographically adjacent samples from the training set. For that, we developed 7 different clustering approaches (see Method details). Methods varied in cluster sizes (10–40 clusters), whether clusters had equal (methods 1–2) or unequal numbers of samples (methods 3–7), and the number of samples per cluster. The median TPS accuracies of the 7 analyses ranged from 636 to 887 years (Tables S4B–S4J). These results demonstrate that TPS has a limited reliance on the temporal components of geographically adjacent samples to achieve accurate dating predictions.

### Phenotypic traits are connected to the TIMs

**Figure 7:**
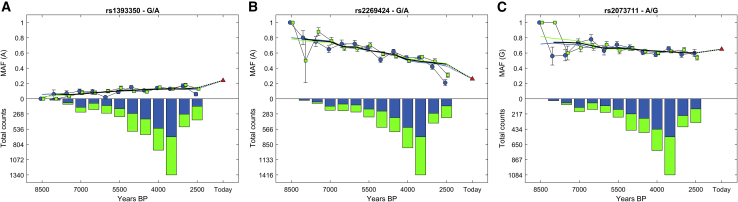
Temporal variation in the allele frequencies of 3 TIMs Bars show the number of individuals genotyped for that TIM. Lines show the centered moving averages of the minor allele frequencies (MAFs) over time and the SEs. Blue refers to radiocarbon-dated samples and green refers to TPS-dated samples. The black line shows the weighted average of the 2 MAF measures. (A) rs1393350 (G/A) in the TYR gene involved in pigmentation. (B) rs2269424 (G/A) is adjacent to the PPT2 and EGFL8 genes associated with immunity. (C) rs2073711 (A/G) is in the CILP gene.

It is often of interest to trace the changes in allele frequencies over time. Here, TPS can be used to "rescue" poorly dated samples to bolster the sample size and power of such analyses. TIMs, such as rs1393350, have been associated with phenotypes, such as those harbored in the HERC2, OCA2, and TYR genes involved in skin, eye, and hair pigmentation (Figure 7A). At least since the Mesolithic, these traits were, reportedly, under selective pressure in favor of variants associated with lighter pigmentation (Olalde et al., 2014; Wilde et al., 2014). rs2269424 (G/A) is another TIM adjacent to the PPT2 and EGFL8, genes associated with immunity. This marker was reported to be under strong selection (Broushaki et al., 2016; Mathieson et al., 2015).

Our temporal trends (Figure 7B) support these findings and the presence of negative selection (Figure 7B). TIM rs2073711 (A/G) (Figure 7C), located in the CILP gene, was reported to be associated with cartilage scaffolding (Wang et al., 2016). Further research is necessary to understand what factors shaped the incline or decline trends of the allele frequencies. Overall, we demonstrated that alleles of poorly dated samples, "rescued" by TPS, yield consistent trends to alleles of radiocarbon dated samples.

## Discussion

Only 48% of the ancient human genomes in the latest AADR release (V50) have a strict direct radiocarbon date (IntCal19, IntCal20, or SHCal20). The remaining samples are imprecisely dated, mostly using archeological context and various estimates. The absence of a reliable alternative to radiocarbon dating is a challenging problem in paleogenomics, which relies on dates to study genomic data. Moreover, although radiocarbon dating is widely accepted as the benchmark standard for dating ancient remains (Mellars, 2006; Ramsey, 2008), its reliance on large amounts of organic material renders many samples undatable and thus unstudied. Radiocarbon dating is also exposed to various environmental biases that decrease its accuracy (Korlevic et al., 2018).

By contrast, genomic dating relies solely on the DNA sequence, making it possible to date remains whose radiocarbon dating cannot be established directly, are in doubt, or are absent. Motivated by our observations that allele frequencies show temporal variability over time, we introduced the concept of TIMs and demonstrated their usefulness to genomic dating. For example, we showed that TIMs associated with traits have increased or decreased their frequency over time, as reported elsewhere (Broushaki et al., 2016; Günther et al., 2018; Hofmanová et al., 2016; Mathieson et al., 2015) and can be used as biomarkers for specific periods. We defined temporal components as aggregations of allele frequency profiles that peaked in specific periods throughout history and modeled genomes as consisting of eight temporal components. We then developed the TPS, an SML tool that converts genomic information into dates. TPS uses random forest regression, which trains on the temporal components of thousands of ancient and modern genomes dating from the Late Mesolithic to modern times and learns how to predict their ages. We demonstrated the accuracy of TPS by showing its ability to correctly predict 3,591 ancient Eurasian skeletons and 1,307 modern individuals, including 731 family members from 130 ancient and 145 modern families. We showed that TPS is robust to incorrect data, noise, and missing data. We further demonstrated its ability to resolve conflicting findings in the literature and increase the power of association studies by dating ancient samples that lacked reliable dates.

TPS is a powerful instrument in the growing paleogeneticist toolkit that can be used when dating is unavailable or in doubt and addresses contradictory findings in the paleogenomic literature. The advantage of adopting an admixture scheme to derive the temporal components over alternative techniques such as linear regression is its resilience to missing data, common to aDNA data. One of the strengths of random forest over dating based on sequence similarity is finding a consensus of correct dates and reducing the effect of outliers and incorrect radiocarbon dates. As an SML algorithm independent of physical measure, TPS can also identify incorrect radiocarbon dates. This is a major advantage for TPS since radiocarbon dating is a physical measure independent of previously dated samples, which is not informed by experience, whereas SML algorithms become more accurate with the increase in sample size (i.e., learning opportunities).

Overall, TPS can be used to date samples, evaluate dated samples, detect outliers or misdated samples, and develop alternative hypotheses to other dating techniques. We envision that genomic dating will become even more accurate with the increase in the number of sequenced populations over time. Therefore, our results should be considered a lower bound to the full potential genomic dating. We note that TPS is neither comparable to genetic dating methods based on measuring the level of Neanderthal inbreeding nor suffers from their biases (Moorjani et al., 2016; Prüfer et al., 2021).

### Limitations of the study

As with all machine learning methods, TPS requires a large training dataset to yield accurate predictions. This limits its applicability to humans and a few farm animals with the large availability of ancient genomes. In humans, TPS is further limited to samples dated from the past 10,000 YBP, where sufficient genomes are available. We note that all of the reported dates off by more than 1,000 YBP were for older samples, for which the training data are sparse. For the same reason, TPS may be limited to Eurasians due to the high availability of their ancient genomes. We also showed that the accuracy of TPS decreases for unsampled periods. This limitation can be resolved when more data are available. Compared to physical measures that are relatively independent of past analyses, the ability of TPS to learn from experience is its advantage—and its weakness, since it may incorporate incorrect data into its model. Solving dating conflicts in the literature (e.g., among relatives) to improve the results without clear traces, as done in the AADR, is a form of genomic photoshopping that poses a challenge for TPS that trains on these data and could resolve such conflicts itself. Finally, we showed that genomes with a wide age range pose difficulties in TPS dating. This limitation may also be resolved when more data are available.

## STAR★Methods

### Key resources table

**Table:** 

REAGENT or RESOURCE	SOURCE	IDENTIFIER
**Deposited data**		

Allen Ancient DNA Resource (AADR) V44.3	David Reich Lab	https://reichdata.hms.harvard.edu/pub/datasets/amh_repo/curated_releases/index_v44.3.html
Allen Ancient DNA Resource (AADR) V50	David Reich Lab	https://reich.hms.harvard.edu/allen-ancient-dna-resource-aadr-downloadable-genotypes-present-day-and-ancient-dna-data
1000 Genomes Phase 3 Project	(Auton et al., 2015)	https://www.internationalgenome.org/data/

**Software and algorithms**		

ADMIXTURE v1.3.0	(Alexander et al. 2009)	http://dalexander.github.io/admixture/download.html
PCA	Matlab	https://se.mathworks.com/help/stats/pca.html
PLINK v1.9	(Chang et al., 2015)	https://www.cog-genomics.org/plink/
Python (v3.0)	Python Software Foundation	https://www.python.org/
scikit-learn	(Pedregosa et al., 2011)	https://scikit-learn.org/stable/
Pandas	(McKinney 2010)	https://pandas.pydata.org/
Temporal Population Structure (TPS)	This study	https://doi.org/10.5061/dryad.s1rn8pkbk

### Resource availability

#### Lead contact

Further information and requests for resources and reagents should be directed to and will be fulfilled by the lead contact, Eran Elhaik (eran.elhaik@biol.lu.se).

#### Materials availability

No biological material was used in this study.

### Method details

#### Curating the ancient genomic dataset

Genotype, dating, and relatedness information for all ancient samples were obtained from the Allen Ancient DNA Resource (AADR) (V50) (https://reich.hms.harvard.edu/allen-ancient-dna-resource-aadr-downloadable-genotypes-present-day-and-ancient-dna-data), a uniformly curated dataset with genotypes and metadata. We constructed a dataset of ancient and modern samples (Figure 1A) by curating 5,563 ancient Eurasian genomes dated between 14,000 and 90 yBP from the AADR. We also obtained 1,307 Eurasians from the 1000 Genomes database (Auton et al., 2015) (Table S1). We retained 147,229 SNPs with the least missingness genotypes for ancient genomes (Esposito et al., 2018). Modern sample ages were set to 10 BP. Because the age of ancient samples is described as a range, we used the mean date obtained from the samples’ annotation data throughout the paper. Files were processed using PLINK v1.9 (Chang et al., 2015).

### Quantification and statistical analysis

#### Constructing the temporal components

To identify the *temporal components*, we randomly selected 300 ancient Eurasians and 50 modern samples from each of five present-day 1000 Genomes populations (Chinese [CHB], Yoruba [YRI], Finnish [FIN], British [GBR], and Tuscan [TSI]). We next applied *unsupervised* ADMIXTURE (v1.3.0) (Alexander et al., 2009) for nine *K’s* [4,12]. Samples were sorted by age (Figure S2A). For each plot, we selected putative *temporal components* that exhibited a temporal characteristic (not geography), i.e., components that were evenly distributed in all samples within a certain period. Initially, ten and three putative modern and ancient components were identified, respectively, with a clear split between the ancient and modern components. Using ADMIXTURE’s allele frequencies output (*p*-file), 15 synthetic samples associated with each *temporal component* candidate were generated for each component (Elhaik et al., 2014; Esposito et al., 2018).

We continued refining the ancient putative components by plotting the primary two principal components of the 150 ancient synthetic samples. The scatter plot showed two overlapping. After applying *supervised* ADMIXTURE, as in Elhaik and Ryan (2019), to the 300 ancient samples with respect to the 135 synthetic ancient ones, we dropped four more components that did not show temporal trends, retaining five ancient *temporal components* (Figure S2B and Table S2). For the modern components, we found that three components best described our samples. When merging them with the ancient samples, these final eight components had minimum noise, smooth profiles, and high ancient-modern sample separation (Figure S2C). We calculated the eight *temporal components* for all the samples using *supervised* ADMIXTURE with respect to the synthetic samples. The datasets had no missing values.

#### Identifying Time Informative Markers (TIMs)

Considering the final *temporal components* (*supervised* ADMIXTURE’s *q*-file), non-random temporal trends were observed over time (Figure 3), suggesting that it is feasible to associate the temporal components with samples’ ages and identify SNPs that contribute to the temporal trends using the per-SNP allele frequencies (*supervised* ADMIXTURE’s *p*-file) (Figures S2B and S4). Sorting the five ancient *temporal components* from old to recent, we used the allele frequencies of each *temporal component* (Table S2) to detect SNPs whose allele frequencies show directed behavior over time. For that, we constructed a time series with the date ranges assigned to the temporal components in 500-year bins (from 10,000 to 0 yBP), resulting in 21 data points (Figure S4). Overlaps in the assigned date ranges of the *temporal components* were averaged to construct the time series. The resulting temporal trends were smoothed using a moving average filter to reduce noise. A total of 62,371 SNPs showing global increasing or decreasing trends or displaying local behavior over sub-intervals of at least 3,000 years were considered TIMs (Figure 3 and Table S2). For a null model, we randomly sampled from the remaining SNPs an equal-sized dataset, considered non-TIMs. We last calculated the eight *temporal components* for all the samples using *supervised* ADMIXTURE with respect to the synthetic samples using all the marker, TIM, and non-TIM sets. These datasets had no missing values.

#### Converting the temporal components to genomic dates

Assuming a dataset (Table S1) where rows represent samples and columns represent the *temporal components* with age as the target variable to be predicted – we developed the Temporal Population Structure (TPS), a supervised machine learning (SML) algorithm that employs a random forest regression (Breiman, 2001) to predict dates from *temporal components*. Additional data like country, dating method, and date standard deviation, were not used for date prediction. TPS calibration consists of four major steps: Preprocessing, learning, evaluation, and prediction (Harrison, 2019, P. 9). TPS was coded in Python (v3.0) using scikit-learn (Pedregosa et al., 2011) and Pandas (McKinney, 2010) libraries.

##### Preprocessing

To identify ancient outliers in the dataset, we plotted the age distribution of the samples and used maximum likelihood estimation to estimate the Gaussian distribution fitting. The distribution was moderately skewed (skewness 0.6 7∈ (0.5,1)) (Figure S1A). Preprocessing consists mainly of data cleaning, checking for missing values, and normalizing the data (García et al., 2015, P. 10). For the first steps, we removed i) 66 samples with a mean age of more than 10,000 YBP and ii) 1,235 samples whose dating annotation was not radiocarbon (Direct: IntCal20) nor archeological context (Context: Archaeological–Period). Overall, 4,158 ancient samples were retained. We next normalized the data by calculating the Z-score for all the features and removed 567 outliers whose Z-score was ≥3 or <=-3 (Ozdemir and Susarla, 2018, P. 93) and whose standard deviation of the date was more than 400 years. The remaining dataset of 3,591 samples had a skewness of 0.23 ∈ (-0.5,0.5) and an approximately symmetric distribution (Figure S1B). Overall, 3,591 ancient and 1,307 modern samples were further analyzed.

##### Feature engineering (feature creation or selection)

Features are independent numeric variables that describe the *temporal components* used as input for the SML model. To increase the number of features, we adopted simple mathematical operations (Rogel-Salazar, 2018, Pp. 100–102) to include 1) the mean of all ancient *temporal components*, 2) the absolute difference between the mean of the first ancient component (calculated for all samples) and the first ancient component, and 3) the sum of the first ancient component and three times the column added in 2), which substitute the first ancient component.

##### Learning

To train the SML on these ten features, we split the ancient samples in a stratified fashion into ten roughly equal-sized subsets based on the mean date and country. The modern samples were stratified into ten roughly equal-sized subsets based only on the country as age was set to 10 BP. A small fraction of the ancient samples (63 samples (<1%)) that could not be matched by date and country appeared only in the training set. Next, the ten ancient subsets were collapsed into two groups stratified as follows: 85% (training and validation) and 15% (unseen or test). For modern samples, the same process was executed. The training set of the ancient samples was combined with the training set of modern samples. Similarly, the testing set of ancient samples was combined with that of the modern samples. Overall, both ancient and modern samples from different ages and locations were included in both the training and testing sets. The combined training set was reshuffled. We then split the training set into 90% (training) and 10% (validation). The training was performed using 10-fold cross-validation. We employed random forest regression that uses ensemble learning for regression with a maximum number of 20 trees (Breiman, 2001). In this procedure, the training dataset is divided into 10 subsets. A holdout method is then repeated 10 times so that each time one of the subsets is used as the validation set, and the remaining nine are combined to form a training set. The random forest algorithm is trained on the nine training sets against the single validation set, evaluating different models that maximize the dating prediction accuracy from the input data and selecting the best model by the vote of the majority of all the trees. Of these ten replicas, a random model is selected as the final model.

##### Predicting the age of undated samples

To date a sample (Figure 2B), the TPS model should be provided with the *temporal components* of the test samples, which are calculated by applying *supervised ADMIXTURE* to its genomic data against the synthetic *temporal components* available from datadryad.

##### Evaluation and prediction

We applied the process above to several cohorts. First, we TPS-dated 740 random samples (15% of the data) stratified over time and space (Figures S5I and S5J) after training the model on the remaining dataset. Second, we TPS-dated all the samples by retaining one sample, at a time, in the unseen set (Figure 7). We carried out this analysis on the entire SNP set, TIMs (Figures S5C and S5D), and non-TIMs (Figures S5A and S5B). Third, families were dated separately, with all the family members as unseen. Likewise, to date the Brandysek individuals, all the Brandyseks were held together in the unseen set. To calculate the standard deviation, standard error, and 95% confidence intervals of the age per sample, we considered each sample at a time as unseen, resampled 90% of the training dataset, retrained the model, dated the unseen sample ten times, and calculated the statistics on the outcome.

#### Evaluating the accuracy of dating predictions

The accuracy of dating prediction was evaluated by comparing the sample’s predicted date (always calculated as unseen data) with its mean published date. Typically, this was assessed using linear regression with significance calculated using *T*-test. To evaluate TPS performances to noisy data, we introduced noise with varying levels of 1%, 10%, and 100% to 1%, 10%, and 100% of the unseen samples. The noise affected all the *temporal components* of the unseen dataset as follows: *Temporal components* within 0.9 quantiles were randomly either increased or decreased by a random value selected from a uniform distribution [0,1]. The noise level represented the proportion of *temporal component* values modified by this procedure. We also evaluated the accuracy of the TPS model by applying the model to an equally-sized dataset of principal components, calculated using Matlab’s pca.m function, by projecting the ancient DNA samples onto the top ten principal components defined by modern-day populations and to an equal-sized dataset with random numbers. When testing family relatives, we evaluated the accuracy against the radiocarbon date and, when unavailable, archeologically-derived date. Additionally, we calculated the predicted age difference among family members, which should be small, regardless of the dating method. Significance was assessed with the two-sided Wilcoxon rank-sum test.

We evaluated the effects of geography on TPS using seven geographical analyses. In each analysis, samples were split between clusters identified based on the similarity of their geographical coordinates using *K*-means clustering so that the seven analyses had: 15 clusters (10-fold cross-validation), 40 clusters (10-fold cross-validation), 20 clusters (10-fold cross-validation), 30 clusters (10-fold cross-validation), 30 clusters (5-fold cross-validation), 15 clusters (10-fold cross-validation), and 10 clusters (10-fold cross-validation). Cluster size varied in the first two methods and was similar in the latter five. The following procedure was applied for all the methods: Following the removal of 22 samples with invalid coordinates, the samples of each cluster were considered unseen, and TPS was trained using the remaining clusters using 5 to 10-fold cross-validation to predict the unseen samples. This process was repeated until all the clusters were predicted. The samples were then divided into a fixed number of 1018 groups based on their latitude and longitude, and their mean accuracies were calculated. The medians of the mean accuracy of each method were then calculated. For example, in the fourth analysis, the dataset was divided into 30 geographical clusters with 100-200 samples per cluster (Figure S7). Then, each cluster was considered unseen at a time, and the model was trained on the remaining clusters using 10-fold cross-validation.

## Data Availability

This paper analyzes existing, publicly available data. These datasets are listed in the key resources table. All original code has been deposited at datadryad and is publicly and freely available as of the date of publication. The DOI is listed in the key resources table. All additional information required to reanalyze the data reported in this paper is available from datadryad.

## Supplemental information

Document S1. Figures S1–S7

Table S1. Ancient DNA dataset of 961 Eurasian individuals used to construct, calibrate, and test TPS, related to Figure 2 The database consists of radiocarbon- and archeology-dated samples.

Table S2. Temporal components datasets Temporal proportions, related to constructing the temporal components, obtained for (S2A) 300 ancient samples for the determination of the 5 ancient temporal components; (S2B) 602 radiocarbon-dated samples with 5 temporal components; (S2C) 300 ancient samples for the determination of the 5 ancient and 3 modern temporal components. Key markers (S2D) *p*-file output by ADMIXTURE with the allele frequencies associated with the 5 ancient components; (S2E) Corresponding Plink fam file; (S2F) time series obtained for the TIMs; (S2G) SNP IDs of non-TIMs.

Table S3. Evaluating the accuracy of TPS and predictions, related to Figure 5B (S3A) TPS results for ancient and modern samples from the Eurasian database (Table S1), with 8 temporal components calculated for all markers. Columns include sample ID, mean date and standard deviation of the published data (BP), TPS statistics: predicted date (BP), and the difference between TPS and the published data—all in years. (S3B) TPS results for ancient and modern samples from the Eurasian database (Table S1), with 8 temporal components calculated for non-TIMs. Columns include sample ID, mean date and standard deviation of the published data (BP), TPS statistics: predicted date (BP), and the difference between TPS and the published data—all in years. (S3C) TPS results for ancient and modern samples from the Eurasian database (Table S1), with 8 temporal components calculated for TIMs. Columns include sample ID, mean date and standard deviation of the published data (BP), TPS statistics: predicted date (BP), and the difference between TPS and the published data—all in years. (S3D) TPS results for dating family relatives. Columns include sample ID, family ID, the 8 temporal components, country of origin, dating method (Direct: IntCal20 or Context: Archaeological), mean published date (BP) and the full date, and finally, TPS predicted date (BP) and the difference from the mean published date—all in years. (S3E) TPS results for 740 ancient samples from the Eurasian database (Table S1), with 8 temporal components calculated for all markers. Columns include sample ID, mean date and standard deviation of the published data (BP), TPS statistics: predicted date (BP), and the difference between TPS and the published data—all in years. (S3F) TPS results for ancient and modern samples from a random matrix of 4,898 × 10 with random values [0,100]. Columns include sample ID, mean date, TPS statistics: predicted date (BP), and the difference between TPS and the published data—all in years. (S3G) TPS results for ancient and modern samples with 10 PCs. Columns include sample ID, mean date and standard deviation of the published data (BP), TPS statistics: predicted date (BP), and the difference between TPS and the published data—all in years.

Table S4. TPS results for the clusterning analyses (S4A) TPS median prediction accuracy of sample ages when various noise levels [1,100%] are introduced to the features of 7–740 unseen samples, related to Evaluating the accuracy of dating predictions. The results are reported in median years for the 740 random samples representing all of the ages and countries in the dataset and should be contrasted with the base median accuracy. For example, when 10% of the test samples are exposed to 10% noise applied to their features, the prediction inaccuracy for the cohort increases only by 12 years. (S4B–S4I) TPS accuracy for geographically adjacent samples. Geographical clusters were either uniformly dispersed in space (with an unequal number of samples) or harbored a similar number of burial sites (with a roughly equal number of samples). For example, in method 1, 15 clusters were created uniformly across Eurasia. Cluster allocation was done based on the fitting of the burial site to the cluster. For comparison reasons, samples were regrouped into 1,018 groups based on their geographical coordinates. The table contains the accuracy results for all of the clusters and each of the 1,018 groups. A detailed description of the clusters used for each method is provided.

Document S2. Article plus supplemental information
