# Supplementary material for: Temporal population structure, a genetic dating method for ancient Eurasian genomes from the past 10,000 years
Source: Cell Rep Methods. 2022 Aug 22;2(8):100270. doi: 10.1016/j.crmeth.2022.100270 (PMC9421539; doi:10.1016/j.crmeth.2022.100270)
Supplement: Document S1. Figures S1–S7 [file mmc1.pdf]

Cell Reports Methods, Volume *2*

Supplemental information

Temporal population structure, a genetic dating method for ancient Eurasian genomes from the past 10,000 years

Sara Behnamian, Umberto Esposito, Grace Holland, Ghadeer Alshehab, Ann M. Dobre, Mehdi Pirooznia, Conrad S. Brimacombe, and Eran Elhaik

Supplementary information

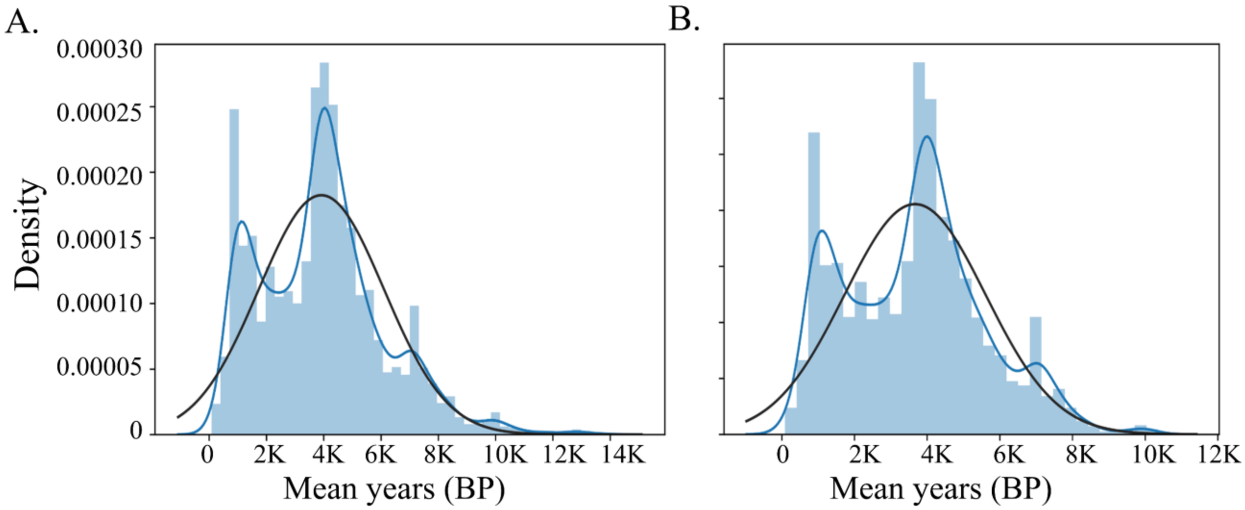

Fig. S1. The frequency distributions and the maximum likelihood Gaussian distribution (black) fit of the ancient sample mean ages (BP) (*x*-axis) before (A) and after (B) outlier removal, related to Preprocessing. Sample mean ages range from 10 to over 14,000 years BP.

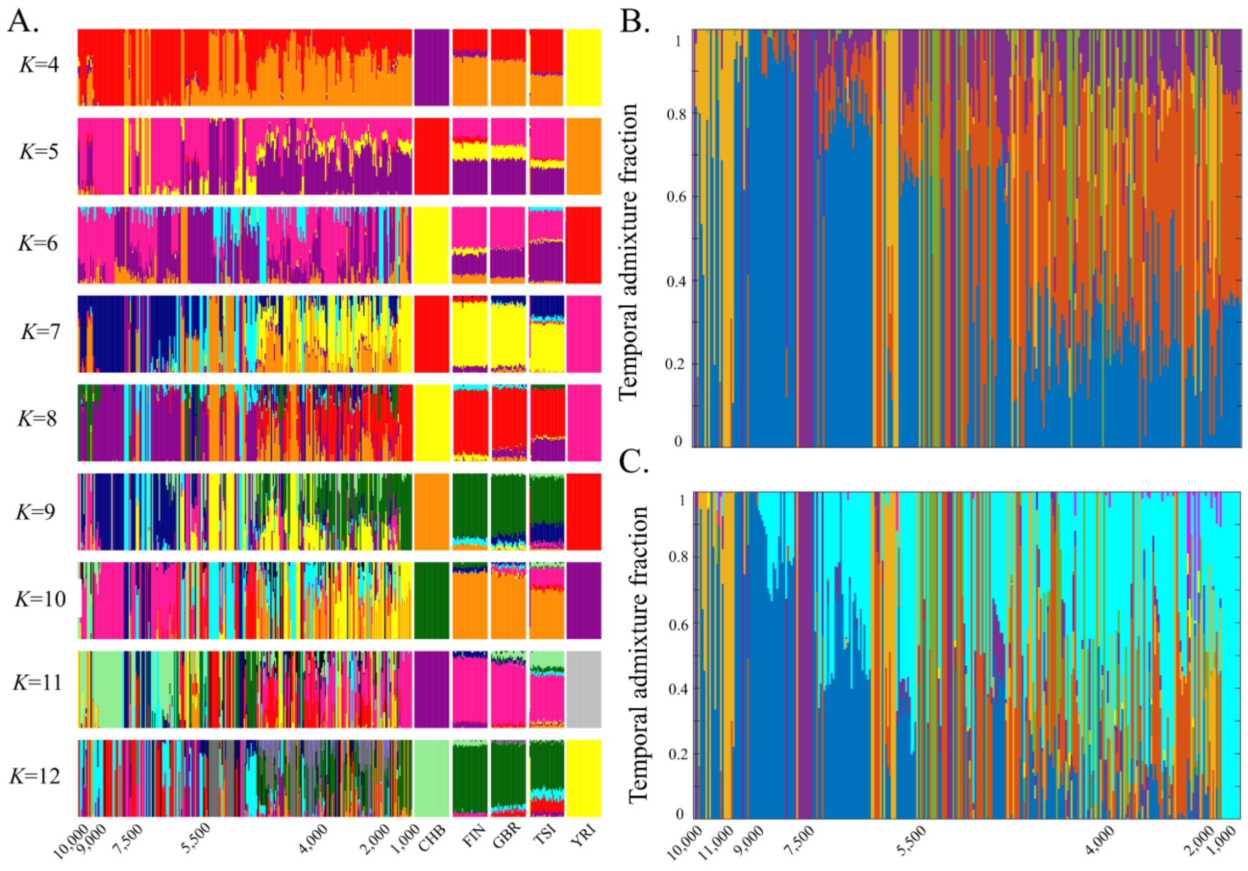

Fig. S2. ADMIXTURE bar plots, related to *Constructing the temporal components*. A) ADMIXTURE bar plots for 300 random ancient samples sorted by age (in BP) and 250 modern samples. The plots were obtained using an *unsupervised* ADMIXTURE's q-file with *K's,* ranging from 4 to 12. Each vertical stacked bar represents an individual. Colors correspond to different components. (B-C) ADMIXTURE bar plots showing the ancient *temporal components* of 300 ancient samples sorted by age (in BP). Results were obtained using *supervised* ADMIXTURE with five ancient (B) and five ancient and three modern (C) *temporal components*. Each vertical stacked bar represents an individual. Colors correspond to the temporal components.

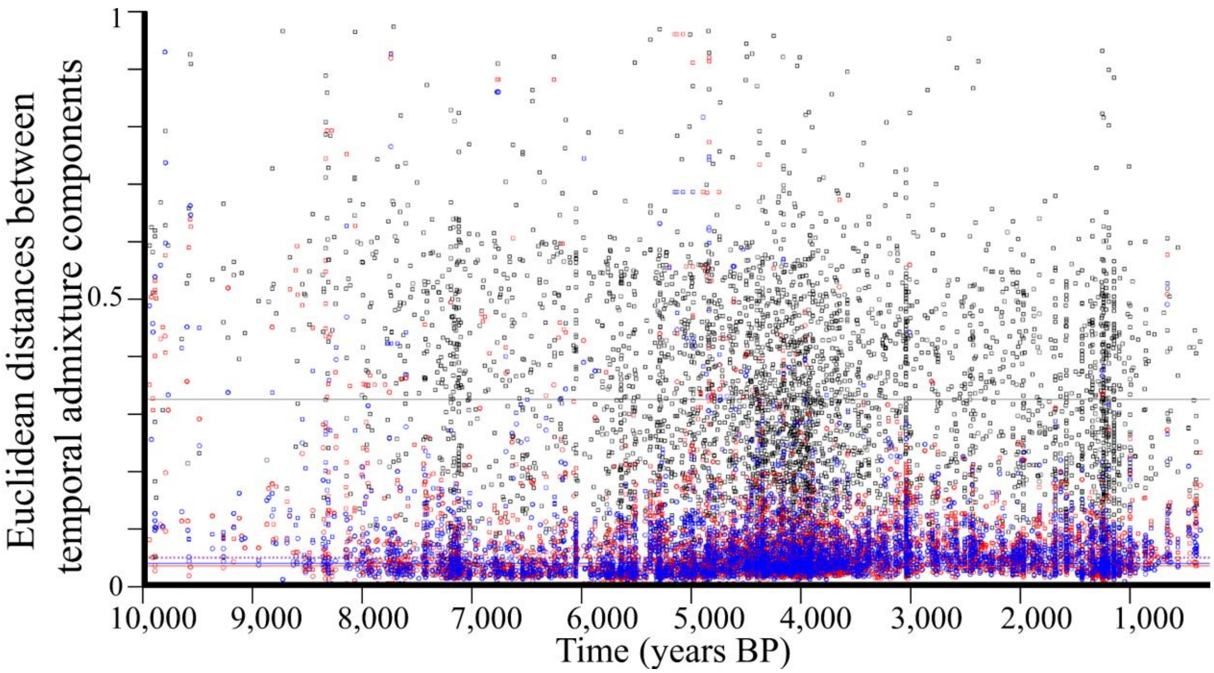

Fig. S3. A scatter plot of the temporal distances between ancient samples of comparative ages (related to Fig. 2). We calculated the Euclidean distance between each sample and its two closest samples within 0-200 (red circles) and 201-500 (blue circles) years and a random sample (black squares) using their eight temporal components. The median of each measure is shown as a straight horizontal line ($n_{0\text{-}200}$=4,158 $\tilde{x}$=0.031; $n_{200\text{-}500}$=4,158 $\tilde{x}$=0.036; n_{random}=4,158 $\tilde{x}$=0.33). We then repeated the analysis by calculating the distances between the two closest samples that were not from the same country and plotted the distances: 0-200 years (red squares) and 0-500 years (blue squares). The median of each measure is shown as a dashed horizontal line ($n_{0\text{-}200}$=4,158 $\tilde{x}$=0.048; $n_{200\text{-}500}$=4,158 $\tilde{x}$=0.046). These results demonstrate the high similarity in the temporal components of ancient samples of similar ages and the independence of those distances from geography (and ancestry). This property enables predicting ages based on the similarity between the temporal components, whether or not geography is considered. We also note the existence of outliers (on average, 0.83% of the closest pairs had a distance higher than random pairs due to incorrect dating, corrupt data, or inaccuracy of the model).

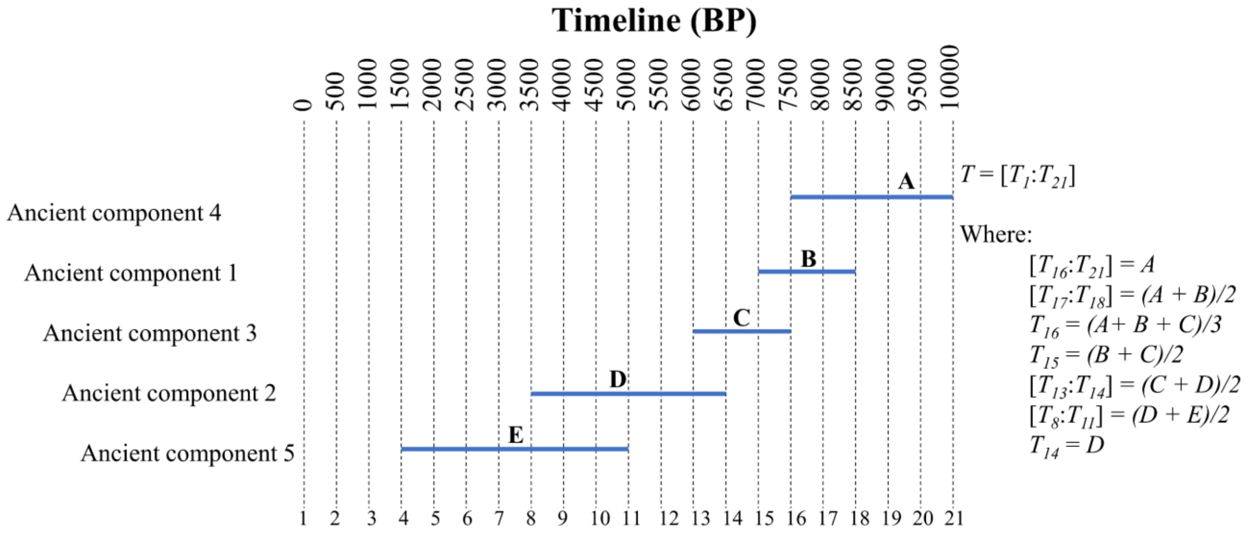

Fig. S4. Construction of time series to identify Time Informative Markers (TIMs) for 1000 random ancient individuals, Related to *Identifying Time Informative Markers (TIMs), and* Table S2F. The five ancient temporal components (Fig. S2B) were assigned a period in our timeline, as shown by the five horizontal bars. The timeline was divided into 21 bins of 500 years. For each SNP, we constructed a time series $T = [T_1{:}T_{21}]$ that consists of 21 allele frequencies (legend). Values were obtained from the allele frequencies of the five ancient temporal components for that SNP, combined as shown in the legend. For instance, the values of T_1 to T_{11} were equal to the allele frequency of ancient temporal component 4 (labeled as A), as this component was dominant during this time interval; the values of T_{12} and T_{13} were the average between the allele frequency of ancient temporal components 4 and 1 (labeled as B), as both components were dominant in this time interval. The final time series was smoothed using a moving average filter to reduce noise. For example, following this procedure, TIM rs6603791 showed a frequency of 0.71 for the 10,000 yBP bin and 0.47 for the 3,000 yBP bin, with decreasing intermediate values. TIMs time series is provided in Table S2.

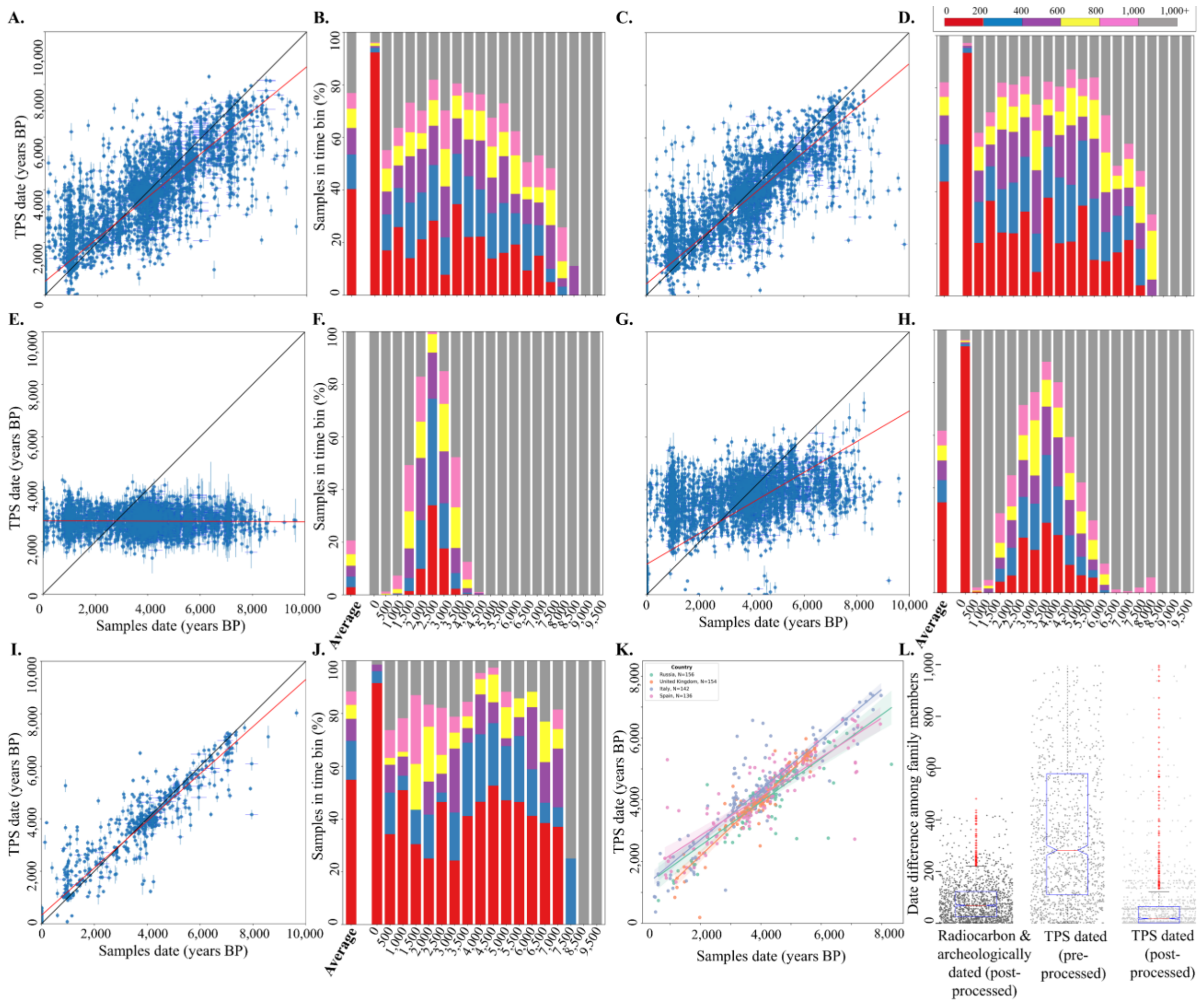

Fig. S5. Evaluating the accuracy of TPS, related to Evaluation and Prediction, and Table S3. Evaluating the accuracy of TPS dating for ancient and modern samples using: 62,371 non-TIM SNPs (t test, n = 4,976, r = 0.9, p = 0) (Figure S5A)(A-B) and TIM SNPs (t test, N = 4,893, r = 0.91, p = 0) (S5C) (C-D) and all the samples, a random matrix of 4,898x10 with random values [0,100] and all the samples (E-F), PCA applied to the full SNPset of all the samples (G-H), and a test set of 740 samples (I-J). Correlation plots show the correlation between TPS and sample dates. Vertical and horizontal bars represent the standard deviation of TPS and radiocarbon dating, respectively. The red line represents the linear fit against the $y = x$ line (black). In all the bar plots, TPS results are aggregated by accuracy. Samples are sorted into 500-year period bins according to their mean dates (BP) (*x*-axis) (e.g., the 4,000 yBP bin represents samples dated from 4,000 to 4,500 yBP). Colors reflect the prediction accuracy, calculated as the difference in years between TPS prediction and the sample date. K) Correlation between radiocarbon and TPS dates for 1076 same-country individuals from four countries with the most samples. TPS dates were distributed along the timeline that follows their radiocarbon dates, confirming that the *temporal components* represent temporal rather than geographical variation. L) Dating differences among 414 ancient same-family members from 130 families for post-processed

published dates ($\tilde{x} = 68$) and TPS pre- ($\tilde{x} = 283$) and post-processed ($\tilde{x} = 17$) dates. The y-axis was limited to 1000 years (full results are in Table S3).

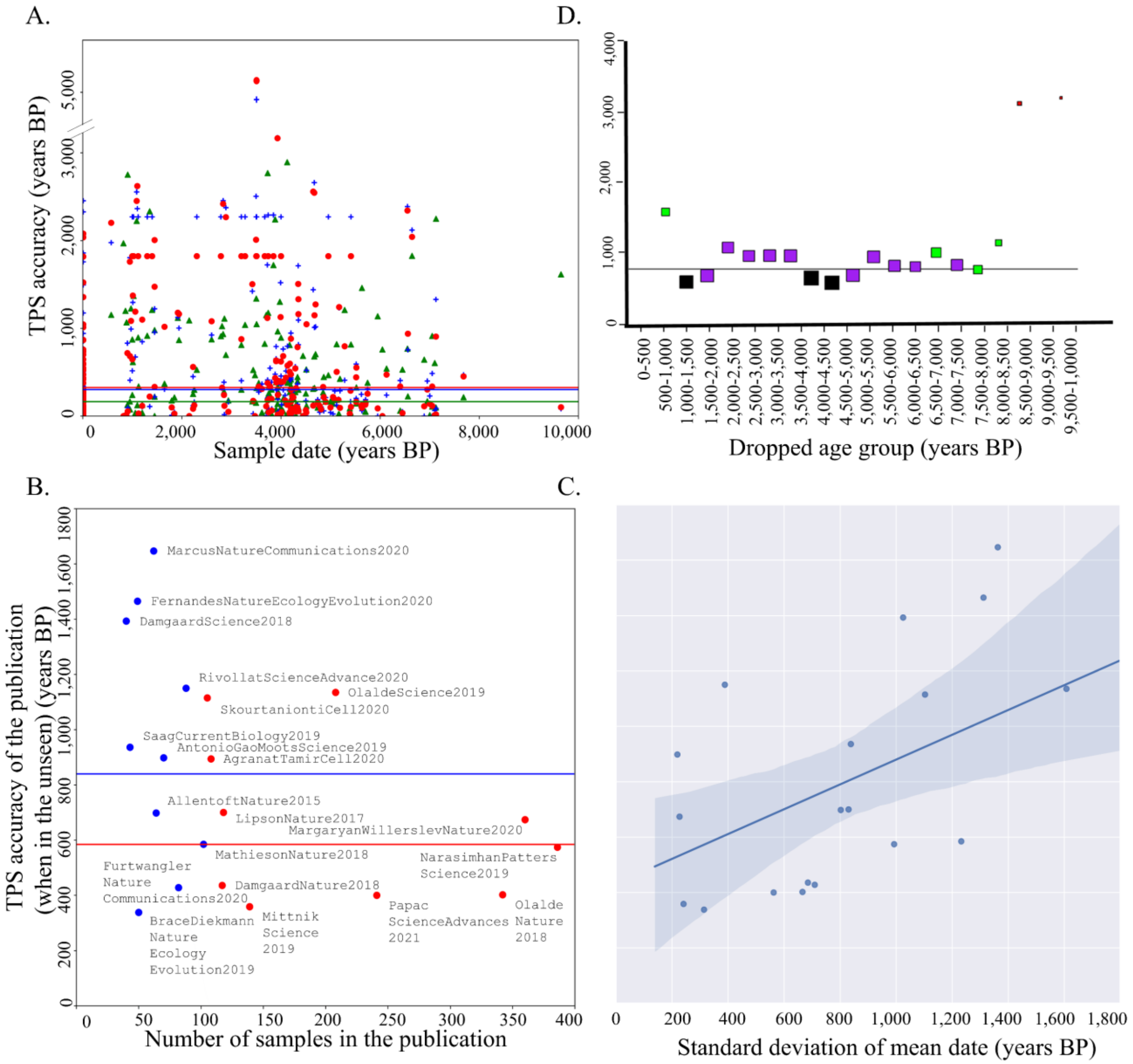

Fig. S6. Evaluating TPS accuracy in various settings, related to *Evaluating the accuracy of dating predictions*. A) TPS accuracy for samples before and after biasing their dates. TPS accuracy is shown for the base samples (green) after adding duplicate samples to the training set and increasing their ages by 1000 years (blue) and after repeating the previous procedure but with 2000 years increase in age (red). For coherency, 30% of the data points were randomly plotted. The median accuracies are shown in color-matched horizontal lines for the entire dataset. Next, TPS accuracy is evaluated for the 20 publications with the most samples (B-C). B) The median TPS accuracy for the samples in each publication is plotted against the sample size of that publication for the top ten (red) and next ten (blue) largest publications. The horizontal lines represent the medians with matching colors for each cohort. Publications are shown in Table S1. C) Linear regression plot of TPS accuracy for each publication against the average standard deviation of the mean date of the samples in that publication ($n = 20$, $r = 0.52$, two-sided t test, t statistic = 2.59, $p = 0.018$). Publications of samples with narrow age distribution were TPS-predicted significantly more accurately than publications that included many samples with a

broad age range. D) TPS median accuracy for predicting ancient samples by age groups without training on that age group. TPS was calculated for all samples grouped by age groups of 500-year-intervals, one at a time, using a training set that excluded same-age-bin samples. Squares show the median accuracy per group. Square sizes and colors are proportional to the number of samples per age group, with red marks for small-sized groups ($n \leq 10$), green marks for medium-small-sized groups ($11 \leq n < 100$), purple marks for medium-sized groups ($101 \leq n < 300$), and black marks for large-sized groups ($301 \leq n < 650$). The weighted mean of all groups is shown in a black horizontal line.

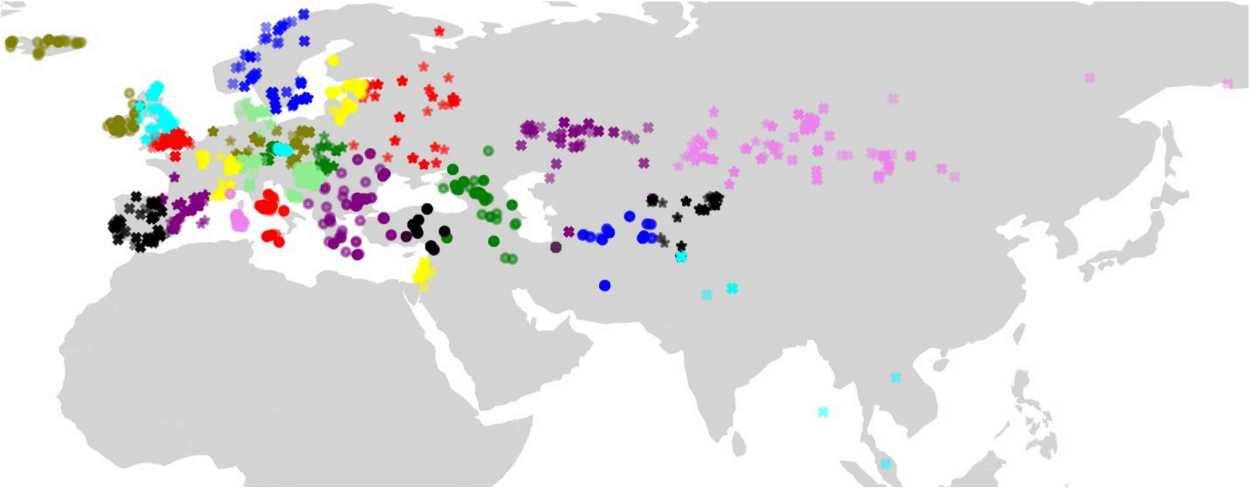

Fig. S7. Evaluation of the effects of geography on TPS using seven geographical analyses, related to *Evaluating the accuracy of dating predictions*. Geographical clusters of ancient samples. 30 Eurasian clusters, each containing 100-200 ancient samples, were used to evaluate the effect of geography on TPS predictions. Clusters are distinguished by colors and markers
